# Heterogeneity of White Matter Hyperintensities in Cognitively Impaired Patients With Cerebral Small Vessel Disease

**DOI:** 10.3389/fimmu.2021.803504

**Published:** 2021-12-09

**Authors:** Tingting Wang, Aoming Jin, Ying Fu, Zaiqiang Zhang, Shaowu Li, David Wang, Yilong Wang

**Affiliations:** ^1^ Department of Neurology, Beijing Tiantan Hospital, Capital Medical University, Beijing, China; ^2^ China National Clinical Research Center for Neurological Diseases, Beijing, China; ^3^ Department of Neurology and Institute of Neurology of First Affiliated Hospital, Institute of Neuroscience, Fujian Medical University, Fuzhou, China; ^4^ Department of Neuroimaging, Beijing Neurosurgical Institute, Beijing Tiantan Hospital, Capital Medical University, Beijing, China; ^5^ Neurovascular Division, Department of Neurology, Barrow Neurological Institute, St. Joseph’s Hospital and Medical Center, Phoenix, AZ, United States; ^6^ Chinese Institute for Brain Research, Beijing, China; ^7^ Advanced Innovation Center for Human Brain Protection, Capital Medical University, Beijing, China; ^8^ Beijing Key Laboratory of Translational Medicine for Cerebrovascular Disease, Beijing, China

**Keywords:** white matter hyperintensities, cognitive impairment, cerebral small vessel disease, heterogeneity, ultrasmall superparamagnetic iron oxide (USPIO)

## Abstract

**Objective:**

Similar white matter hyperintensities (WMH) might have different impact on the cognitive outcomes in patients with cerebral small vessel disease (CSVD). This study is to assess the possible factors related to the heterogeneity of WMH in cognitively impaired patients with CVSD.

**Methods:**

We analyzed data from a cohort of patients with CVSD who were recruited consecutively from the Beijing Tiantan Hospital from 2015 to 2020. WMH, lacunes, enlarged perivascular space (ePVS), microbleeds and lacunar infarcts were rated on brain MRI. A score of <26 on the Montreal Cognitive Assessment (MoCA) indicated cognitive impairment. A mismatch was defined as the severity of WMH not matching the severity of cognitive dysfunction. Type-1 mismatch was defined as a mild WMH (Fazekas score = 0-1) associated with cognitive impairment, and type-2 mismatch was defined as a severe WMH (Fazekas score = 5-6) associated with normal cognitive function. Ultrasmall superparamagnetic iron oxide (USPIO)-enhanced SWI on 3-Tesla MRI was used to image the penetrating arteries in basal ganglia to explore the underlying mechanism of this mismatch. Multivariable logistic regression was used to analyze the association between the imaging features and cognitive impairment.

**Results:**

In 156 patients, 118 (75.6%) had cognitive impairment and 37 (23.7%) showed mismatch. Twenty five (16.0%) had type-1 mismatch and 12 (7.7%) had type-2 mismatch. Regression analysis found that WMH, lacunes, microbleeds and total CSVD scores were associated with cognitive impairment and were independent of vascular risk factors. However, lacunes, microbleeds and total CSVD scores were related to the mismatch between WMH and cognitive impairment (p=0.006, 0.005 and 0.0001, respectively). Specially, age and ePVS in basal ganglia were related to type-1 mismatch (p=0.04 and 0.02, respectively); microbleeds and total CSVD scores were related to type-2 mismatch (p=0.01 and 0.03, respectively). Although the severity of WMH was similar, the injury scores of penetrating arteries were significantly different between those with and without cognitive impairment (p=0.04).

**Conclusions:**

Heterogeneity of WMH was present in cognitively impaired patients with CSVD. Conventional imaging features and injury of penetrating arteries may account for such heterogeneity, which can be a hallmark for early identification and prevention of cognitive impairment.

## Introduction

Cerebral small vessel disease (CSVD) is generally caused by disorders of the intrinsic cerebral arteriolar system (1). CSVD is an important subtype of stroke and the major cause of dementia (2). White matter hyperintensities (WMH) are the most common feature of CSVD on brain magnetic resonance imaging (MRI) (3) with a prevalence up to 94% in the general population aged around 80 (4). It is associated with cognitive decline, a 90% increased risk of dementia and a 200% increased risk of stroke (5).

Several studies have focused on the association between WMH and clinical outcomes. Most of these studies found that extensive WMH burden was usually associated with increased risk of incident stroke, dementia and mortality in general population or in populations at high risk for vascular disease or dementia (6). WMH were often heterogeneous, for example, the volume of WMH could increase, remain stable, or even regress one year after a minor stroke (7–9). The microstructures within WMH, including the integrity of neural fibers, the structure of small vessels and histopathology, might vary distinctly, causing the heterogeneity of WMH (7, 10, 11). However, the effect of such heterogeneity of WMH on clinical symptoms remains uncertain.

Recently, ultrasmall superparamagnetic iron oxide (USPIO)-enhanced susceptibility-weighted imaging (SWI) on 3-Tesla MRI has enhanced the visualization of small vessels (12, 13). The structure and function of supplying vessels could directly reflect the real severity of WMH. Therefore, applying USPIO-SWI to assess the small vessels might identify factors contributing to the heterogeneity of WMH.

In this cross-sectional study of imaging features and clinical outcomes in patients with CSVD, we observed the heterogeneity of WMH severity on cognitive impairment. In order to identify the association of vascular risk factors and imaging features that contribute to this heterogeneity and its potential underlying mechanism, we used USPIO-SWI to evaluate the relevant small perforating arterioles.

## Methods

### Patients

Patients with CSVD were recruited prospectively and consecutively from Neurology Department of Beijing Tiantan Hospital from 2015 to 2020. All patients had a definitive diagnosis of lacunar stroke or headache or dizziness but with typical MRI features of CSVD. To qualify for enrollment, patients must meet one of the following inclusion criteria: 1) patients had lacunar stroke syndrome, and MRI showed a recent subcortical small lacunar infarction responsible for the clinical symptoms. If no such lesion was found on MRI, the clinical definition of lacunar syndromes described by Fisher (14) was used. 2) Patients underwent MRI because of headache or dizziness, although the neurological examination was negative, and had at least one of the typical MRI features of CSVD (2, 15): recent small subcortical infarcts; WMH Fazekas score ≥2; WMH Fazekas score=1, combined with at least one lacune or at least two cardiovascular risk factors (including hypertension, dyslipidemia, diabetes, smoking, or ischemic heart disease).

The exclusion criteria were as follows: 1) cortical infarct on MRI; 2) acute cerebral infarction with a diameter >20 mm on MRI; 3) acute cerebral hemorrhage or subarachnoid hemorrhage within 14 days before recruitment; 4) other WMH disorders, including those with nonvascular origin, e.g., multiple sclerosis and primary or secondary encephalitis; 5) diagnosis of neurodegenerative disease of the central nervous system, such as Alzheimer’s disease, Parkinson’s disease and so on; 6) complications with psychiatric disorders, including depression and anxiety; 7) significant stenosis (>50%) of the cervical or intracranial arteries or possible cardioembolic source (e.g., atrial fibrillation or a valvular prosthesis); and 8) failure to complete all sessions in the prospective study stream.

The diagnosis of CSVD was made by at least two trained neurologists after reviewing the clinical manifestations, MRI features and results of other diagnostic tests.

### Vascular Risk Factors

Baseline data included age, sex, and cardiovascular risk factors such as body mass index (BMI), hypertension, diabetes mellitus, dyslipidemia, ischemic heart disease, current smoking, and alcohol consumption. Hypertension was defined as the systolic blood pressure ≥140 mmHg or the diastolic blood pressure ≥90 mmHg or when the patient was on antihypertensive treatments at the entry. Diabetes mellitus was defined as the fasting blood glucose level ≥120 mg/dL or when the patient was on antidiabetic treatments at the entry. Dyslipidemia was defined as the total cholesterol level ≥240 mg/dL or the high-density lipoprotein measurement< 35 mg/dL or when the patient was on lipid-lowering treatments. Ischemic heart disease was diagnosed by the medical chart-confirmed history. Current smoking was defined as smoking at entry or quitting smoking within the previous year. Moderate to severe alcohol consumption was defined as consuming ≥ two standard alcoholic beverages per day within the last year.

### Brain MRI Acquisition

All patients underwent brain MRI on a 3-Tesla scanner (Siemens MAGNETOM Prisma, Erlangen, Germany) with a Siemens 64-channel Prisma head coil.

#### Plain MRI Scan

The structural MRI included a T1-weighted sequence [repetition time (TR)/inversion time (TI)/echo time (TE) = 2300/900/2.3 ms; field of view (FOV) = 256 × 256 × 196 mm^3^; voxel size = 1.0× 1.0 × 1.0 mm^3^, flip angle = 8°] for anatomic reference and a T2-weighted fluid-attenuated inversion recovery (FLAIR) sequence (TR/TI/TE = 5000/1800/386 ms; FOV = 256×256×196 mm^3^; voxel size = 1.0× 1.0 × 1.0 mm^3^, flip angle = 40°) for assessment of WMH, lacunes and enlarged perivascular space (ePVS). In addition, a diffusion-weighted imaging (DWI) sequence (TR/TE = 6100/65 ms; FOV = 184×226×143 mm^3^; voxel size = 1.6 × 2.0 × 5.0 mm^3^, diffusion sensitizing gradient directions b = 1200 sec/mm^2^) was performed for the detection of new lacunar infarcts, and a SWI sequence (TR/TE = 29/20 ms; FOV = 210×240×159 mm^3^; voxel size = 0.5 × 0.5 × 1.5 mm^3^, flip angle = 15°) was used to detect microbleeds.

#### USPIO-SWI Scan

The SWI sequence was performed after intravenous administration of USPIO (ferumoxytol; AMAG Pharmaceuticals, Cambridge, MA; 3 mg/kg) (12). The SWI images were acquired using a T2*-weighted, 3D-gradient echo sequence: TR/TE = 29/20 ms, FOV = 210×240×159 mm^3^, voxel size = 0.9 × 0.9 × 0.9 mm^3^, flip angle = 15°, number of slices = 176, slice oversampling 18.2%. Blood pressure was measured before the initiation of MRI scanning and within 15 minutes after scanning to monitor any signs of hypertension or hypotension.

### Brain MRI Assessment

#### CSVD Imaging Markers

The presence, location, and lesion size of typical CSVD imaging markers were assessed according to the Standards for Reporting Vascular Changes on Neuroimaging (STRIVE) (3) by two experienced neuroradiologists (Q.H., with 25 years of experience, and S.Y.L., with 3 years of experience) who were blinded to the patients’ clinical information. To assess the WMH severity, we used the Fazekas score (16) for visual assessment and UBO Detector (https://cheba.unsw.edu.au/group/neuroimaging-pipeline) for computational assessment. We combined the periventricular and deep Fazekas scores as the total WMH score (0–6). To conveniently reflect the severity of the WMH, we graded WMH as mild (Fazekas score=0-1), moderate (Fazekas score=3-4) or severe (Fazekas score=5-6) types. UBO Detector is a cluster-based and fully automated WMH extraction pipeline. We followed the previously published technical details to preprocess the structural images. The results of UBO Detector highly correlated with the manually traced results and were significantly associated with Fazekas scores (17). The total CSVD score (range 0-4) was calculated on the basis of the individual imaging features, and points were assigned as follows (18): 1 for any lacune, 1 for any microbleed, 1 for moderate-to-severe ePVS in the basal ganglia (BG-ePVS > 10), and 1 for WMH (deep WMH, Fazekas=2 or 3 and/or periventricular WMH, Fazekas=3). The interclass correlation coefficient for interobserver ratings for WMH was 0.94; lacunes, 0.90; BG-ePVS, 0.85; microbleeds, 0.82; and lacunar infarct, 0.96.

#### Penetrating Arteries Assessment

To assess penetrating arteries in the basal ganglia, USPIO-SWI images were first processed. Raw SWI image data were interpolated to isotropic resolution, and minimum intensity projection (mIP) was created in the coronal and axial views, with an effective slice thickness of 8 mm. The medial and lateral lenticulostriate arteries (LSAs) could be reconstructed in the coronal view according to the previous histology work of Salamon (19). Injury of the LSAs was assessed on an ordinal scale from 0-2 by counting the number of LSAs: 0 points (if 2 visible), 1 point (if 1 visible), and 2 points (if 0 visible). The medial LSAs and lateral LSAs were rated separately by two experienced radiologists who were blinded to the patients’ clinical information and the conventional imaging features. The total score was obtained by summing the 2 partial scores.

### Clinical Outcome

The clinical outcome was defined as cognitive impairment. Considering that the Montreal Cognitive Assessment (MoCA) has a high level of sensitivity and specificity for detecting mild cognitive impairment, we used it to assess global cognitive performance for all subjects. A MoCA score of < 26 indicated cognitive impairment and the education level was accounted for in the scoring (20–22). The previous studies demonstrated that extensive WMH was usually associated with cognitive decline (5, 6). However, we observed a “mismatch” between WMH severity and cognitive impairment, we defined it as the “mismatch type” in present study. Specifically, mild WMH (Fazekas =0-2) with cognitive impairment was defined as type-1 mismatch, and severe WMH (Fazekas =5-6) with normal cognitive function was defined as type-2 mismatch. If on the contrary, are the match types.

### Statistical Analysis

We used χ2 tests and Mann-Whitney tests to test for differences between patients with and without cognitive impairment. We explored the association between CSVD imaging markers and cognitive impairment by multivariable logistic regression analysis, which was adjusted for age, sex, and risk factors that are frequently associated with cognitive impairment, including hypertension, dyslipidemia, diabetes mellitus, smoking and drinking. To explore what factors contributed to the mismatch between WMH severity and cognitive impairment, we performed χ2 tests and Mann-Whitney tests to compare the differences in clinical factors, conventional imaging features of CSVD and injury scores of LSAs between patients with and without mismatch types. The results of regression analysis are presented as odds ratios (ORs) with 95% confidence intervals. All analyses were conducted with SAS software, version 9.4 (SAS Inc., Cary, N.C., USA).

## Results

**Figure 1:**
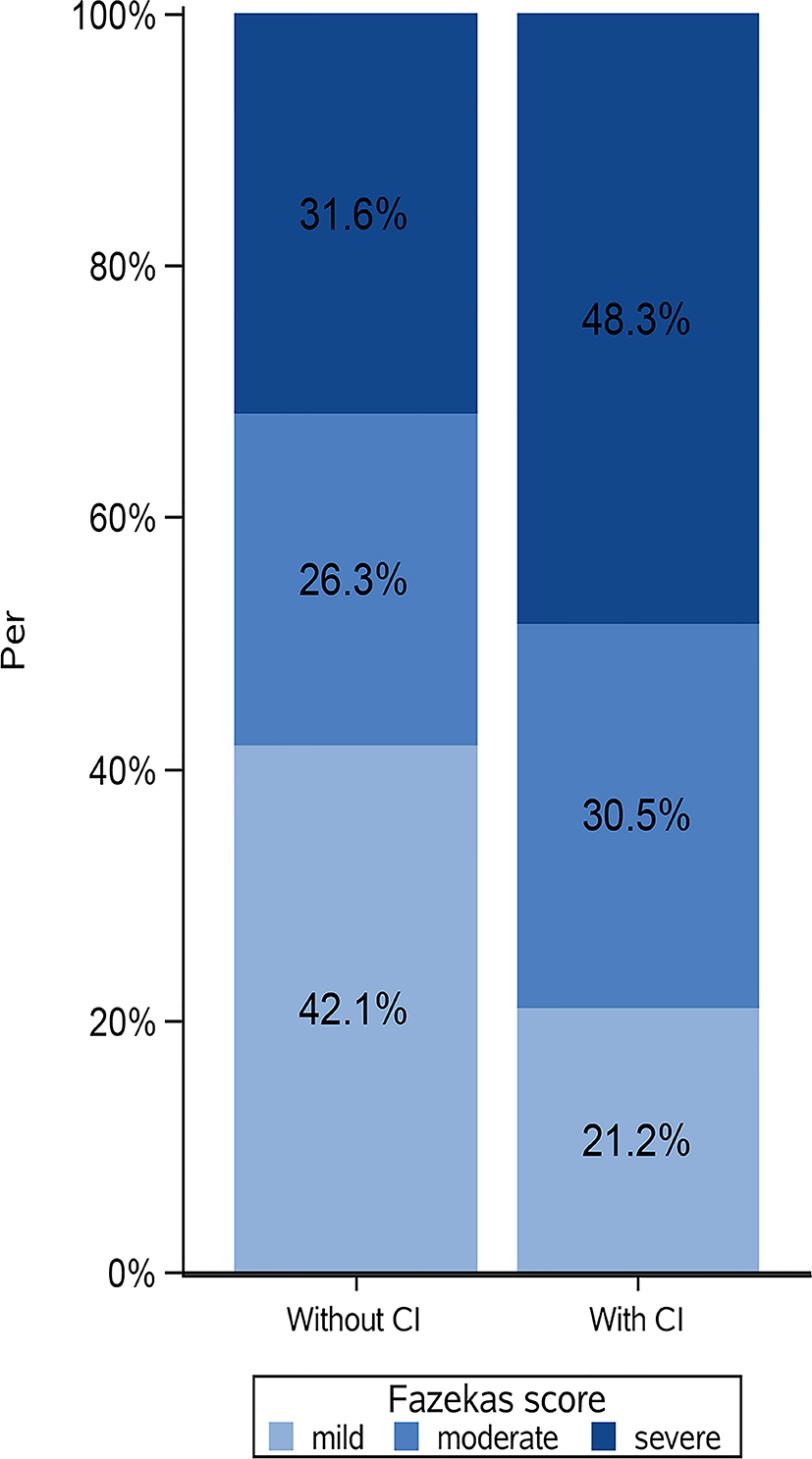
Distribution of white matter hyperintensities (WMH) severity in patients with and without cognitive impairment. CI, cognitive impairment; Fazekas score, mild (Fazekas score=0-2); moderate (Fazekas score=3-4); severe (Fazekas score=5-6). Patients without cognitive impairment could show severe WMH, on contrary, patients with normal cognitive function could show mild WMH.

Of 172 patients in the original cohort, 10 were excluded because of missing MoCA scores, and 6 were excluded because of missing MRI data. Finally, 156 patients were included in this analysis. Among them, 118 patients (75.6%) had cognitive impairment. The median age was 57.0 (50.0-65.0) years. The patients with cognitive impairment were older. Regarding the conventional MRI features, the Fazekas scores and volume of WMH, as well as the presence of lacunes, microbleeds and ePVS, were higher in patients with cognitive impairment than in those without (**Table 1**). The distribution of WMH severity in patients with and without cognitive impairment was showed in **Figure 1**.

**Table 1:** Clinical and radiological characteristics of the study population.

Characteristics	Total patients (n = 156)	With cognitive impairment (n = 118)	Without cognitive impairment (n = 38)	P value
Age, median (IQR), y	57.0 (50.0-65.0)	58.0 (52.0-66.0)	51.5 (43.0-60.0)	0.001
Male, N (%)	92.0 (59.0)	72 (61.0)	20 (52.6)	0.45
BMI, (kg/m^2^)	25.0 (22.5-27.3)	24.9 (22.3-27.4)	25.5 (23.6-27.1)	0.72
Medical history, N (%)				
Hypertension	100 (64.1)	79 (67.0)	21 (55.3)	0.24
Dyslipidemia	53 (34.0)	41 (34.8)	12 (31.6)	0.84
Diabetes mellitus	35 (22.4)	27 (22.9)	8 (21.1)	1.00
Ischemic heart disease	16 (10.3)	16 (13.6)	0	0.01
Current smoking, N (%)	71 (45.5)	55 (46.6)	16 (42.1)	0.71
Alcohol consumption, N (%)	50 (32.1)	36 (30.5)	14 (36.8)	0.55
MOCA total score, median (IQR)	22.0 (16.0-25.0)	19.5 (15.0-23.0)	27.0 (26.0-28.0)	<0.0001
WMH Fazekas score, median (IQR)	4.0 (2.0-6.0)	4.0 (3.0-6.0)	3.0 (2.0-5.0)	0.01
WMH volume, median (IQR)	15.7 (5.7-31.2)	18.5 (7.7-33.3)	9.4 (2.9-16.7)	0.01
MRI markers, N (%)				
Recent small subcortical infarcts	25 (16.0)	19 (16.1)	6 (15.8)	1.00
Lacunes	108 (69.2)	87 (73.7)	21 (55.3)	0.04
Microbleeds	89 (57.1)	79 (67.0)	10 (26.3)	<0.0001
BG-ePVS (N > 10)	114 (73.1)	92 (78.0)	22 (57.9)	0.02
Total CSVD score, (median, IQR)	3.0 (2.0-4.0)	3.0 (2.0-4.0)	2.0 (1.0-3.0)	<0.0001

IQR, interquartile range; BMI, body mass index (kg/m^2^); MoCA, Montreal Cognitive Assessment; WMH, white matter hyperintensities; BG-ePVS, enlarged perivascular space in basal ganglia; CSVD, cerebral small vessel disease.

### Association Between MRI Features and Cognitive Impairment

The severity of WMH, lacunes, microbleeds, ePVS and total CSVD scores were all significantly associated with the cognitive impairment in the unadjusted multivariable logistic regression model (**Table 2**). In addition, compared with mild WMH (Fazekas=0-2), severe WMH (Fazekas=5-6) increased the risk of cognitive impairment by 204% (OR: 3.04, 95% confidence interval: 1.26-7.36), but moderate WMH (Fazekas=3-4) did not (OR: 2.30, 95% confidence interval: 0.90-5.90). After adjusting for age, sex, hypertension, dyslipidemia, diabetes mellitus, current smoking and alcohol consumption, the associations with the severity of WMH, lacunes, microbleeds and total CSVD scores remained significant (WMH: OR: 1.28, 95% confidence interval: 1.01-1.64; lacunes: OR: 2.49, 95% confidence interval: 1.08-5.73; microbleeds: OR: 5.29, 95% confidence interval: 2.13-13.13; total CSVD scores: OR: 1.79, 95% confidence interval: 1.28-2.52). However, the association between ePVS and cognitive impairment became insignificant, and the difference in the risk of cognitive impairment between mild WMH and severe WMH was insignificant. The above results suggested that the severity of WMH, presence of lacunes and microbleeds, and total CSVD scores were associated with the risk of cognitive impairment, which were independent of conventional vascular risk factors. Nevertheless, the risk of cognitive impairment did not increase when increased severity of WMH (moderate WMH: OR: 1.69, 95% confidence interval: 0.60-4.76; severe WMH: OR: 2.41, 95% confidence interval: 0.89-6.55).

**Table 2:** Association between MRI features and cognitive impairment.

Imaging features of CSVD	Unadjusted OR (95% confidence interval)	Adjusted^a^ OR (95% confidence interval)
Fazekas score of WMH		
All	1.36 (1.10-1.69)^c^	1.28 (1.01-1.64)^b^
Mild	reference	reference
Moderate	2.30 (0.90-5.90)	1.69 (0.60-4.76)
Severe	3.04 (1.26-7.36)^b^	2.41 (0.89-6.55)
Lacune	2.72 (1.06-4.86)^b^	2.49 (1.08-5.73)^b^
Microbleeds	5.67 (2.50-12.85)^d^	5.29 (2.13-13.13)^d^
BG-ePVS (N > 10)	2.57 (1.18-5.60)^b^	1.74 (0.72-4.18)
Total CSVD Score	1.94 (1.43-2.63)^d^	1.79 (1.28-2.52)^d^

CSVD, cerebral small vessel disease; WMH, white matter hyperintensities; BG-ePVS, enlarged perivascular space in basal ganglia; OR, odds ratio.

Fazekas score of WMH, mild (Fazekas score = 0-2); moderate (Fazekas score = 3-4); severe (Fazekas score = 5-6).

a Adjusted for age, sex, hypertension, dyslipidemia, diabetes mellitus, current smoking, and alcohol consumption.

b p < 0.05.

c p < 0.01.

d p < 0.001.

### Comparison of Clinical and Imaging Factors Between the Mismatch and Match Types

Among all patients, 37 patients (23.7%) were observed with mismatch types and 73 patients (46.8%) had corresponding matches between imaging findings and cognitive impairment. The presence of imaging features, including lacunes, microbleeds and total CSVD scores appeared significantly different between the mismatch type and match type, which were lower in the mismatch type than in the match type (**Table 3**). However, recent small subcortical infarcts and BG-ePVS did not show any difference between the two types, nor did clinical factors, i.e., age, sex, and vascular risk factors, including hypertension and diabetes mellitus. Our analysis suggested that, compared with clinical factors, a lower rate of imaging features, especially lacunes and microbleeds, and lower total CSVD scores, were more likely to result in a mismatch between WMH severity and cognitive impairment.

**Table 3:** Comparison of clinical and imaging factors between mismatch and match types.

Characteristics	Mismatch type (n = 37)	Match type (n = 73)	P value
Age, median (IQR), y	54 (46–63)	56 (48-65)	0.65
Male, N (%)	24 (64.9)	40 (54.8)	0.41
BMI, (kg/m^2^)	25.6 (23.1-27.2)	25.1 (22.5-28.3)	0.78
Medical history, N (%)			
Hypertension	24 (64.9)	41 (56.2)	0.42
Dyslipidemia	10 (27.0)	25 (34.3)	0.52
Diabetes mellitus	9 (24.3)	11 (15.1)	0.30
Ischemic heart disease	5 (13.5)	4 (5.5)	0.16
Current smoking, N (%)	19 (51.4)	28 (38.4)	0.22
Alcohol consumption, N (%)	12 (32.4)	22 (30.1)	0.83
MRI markers, N (%)			
Recent small subcortical infarcts	7 (18.9)	11 (15.1)	0.60
Lacunes	20 (54.1)	59 (80.8)	0.006
Microbleeds	13 (35.1)	47 (64.4)	0.005
BG-ePVS (N > 10)	27 (73.0)	48 (65.8)	0.52
Total CSVD score, (median, IQR)	2 (1-3)	3 (2-4)	0.0001

IQR, interquartile range; BMI, body mass index (kg/m^2^); BG-ePVS, enlarged perivascular space in basal ganglia; CSVD, cerebral small vessel disease.

Mismatch type: mild white matter hyperintensities (Fazekas score = 0-2) with cognitive impairment, or severe white matter hyperintensities (Fazekas score = 5-6) with normal cognitive function. If on the contrary, is the match type.

### Clinical and Imaging Factors Related to the Two Types of Mismatch

Among all patients with mismatch types, 25 patients (16.0%) had type-1 mismatch and 12 (7.7%) with type-2 mismatch. Although lower rates of lacunes and microbleeds, as well as lower CSVD scores, showed an overall relationship to mismatch types, when examining one mismatch type, the results differed (**Table 4**). Age and BG-ePVS were significantly different between those in the type-1 mismatch group and match group (p=0.04 and 0.02, respectively). The patients in the type-1 mismatch group were older and had higher rates of BG-ePVS than those in the type-1 match group. On the rates of microbleeds and total CSVD scores, the patients in the type-2 mismatch group showed lower rates of microbleeds (p=0.01) and lower total CSVD scores (p=0.03) than those in the type-2 match group. The results of this analysis suggested that in patients with mild WMH, older age and BG-ePVS might increase the risk of cognitive impairment. However, among patients with severe WMH, lacking microbleeds or lower total CSVD scores might decrease the risk of cognitive impairment.

**Table 4:** Clinical and imaging factors related to type-1 mismatch and type-2 mismatch.

Characteristics	Type-1			Type-2		
	Mismatch (n = 25)	Match (n = 16)	P value	Mismatch (n = 12)	Match (n = 57)	P value
Age, median (IQR), y	54.0 (45.0-63.0)	50.0 (40.0-54.0)	0.04	56.0 (46.5-70.5)	58.0 (50.0-68.0)	0.66
Male, N (%)	16 (64.0)	9 (56.3)	0.75	8 (66.7)	31 (54.4)	0.53
BMI, (kg/m^2^)	26.0 (23.1-27.3)	25.2 (22.9-27.4)	0.70	25.6 (23.3-26.6)	25.0 (22.5-28.6)	0.95
Medical history, N (%)						
Hypertension	18 (72.0)	9 (56.3)	0.33	6 (50.0)	32 (56.1)	0.76
Dyslipidemia	5 (20.0)	4 (25.0)	0.72	5 (41.7)	21 (36.8)	0.75
Diabetes mellitus	7 (28.0)	3 (18.8)	0.71	2 (16.7)	8 (14.0)	1.00
Ischemic heart disease	5 (20.0)	0	0.14	0	4 (7.0)	1.00
Current smoking, N (%)	13 (52.0)	6 (37.5)	0.52	6 (50.0)	22 (38.6)	0.53
Alcohol consumption, N (%)	7 (28.0)	5 (31.3)	1.00	5 (41.7)	17 (29.8)	0.50
MRI markers, N (%)						
Recent small subcortical infarcts	7 (28.0)	5 (31.3)	1.00	0	6 (10.5)	0.58
Lacunes	11 (44.0)	7 (43.8)	1.00	9 (75.0)	52 (91.2)	0.14
Microbleeds	9 (36.0)	3 (18.8)	0.31	4 (33.3)	44 (77.2)	0.01
BG-ePVS (N > 10)	18 (72.0)	5 (31.3)	0.02	9 (75.0)	43 (75.4)	1.00
Total CSVD score, median (IQR)	2.0 (1-2)	0.5 (0-1.5)	0.07	3.0 (2.5-3.5)	4.0 (3.0-4.0)	0.03

IQR, interquartile range; BMI, body mass index (kg/m^2^); BG-ePVS, enlarged perivascular space in basal ganglia; CSVD, cerebral small vessel disease.

Type-1 mismatch, mild white matter hyperintensities (Fazekas score = 0-2) with cognitive impairment; type-2 mismatch, severe white matter hyperintensities (Fazekas score = 5-6) with normal cognitive function. If on the contrary, are the match types.

### Rating of Penetrating Arteries Associated With WMH

**Figure 2:**
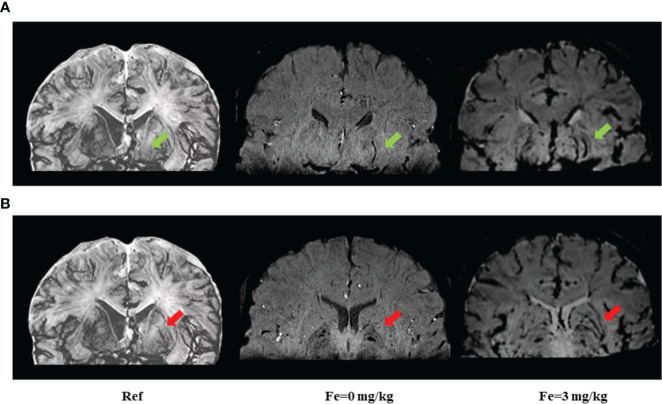
Imaging of medial and lateral lenticulostriate arteries (LSAs) before and after ultrasmall superparamagnetic iron oxide (USPIO) enhanced susceptibility-weighted imaging (SWI) on 3-Tesla MRI. The medial and lateral LSAs were reconstructed in the coronal view of SWI. All images were minimum intensity projected (mIP) with an effective slice thickness of 8 mm. **(A)** green arrows point to the medial LSAs; **(B)** red arrows point to lateral LSAs. Ref: reference pictures were adapted from Salamon’s histology work (19) and could also be available online at Salamon’s Neuroanatomy and Neurovasculature Web-Atlas Resource (http://www.radnet.ucla.edu/sections/DINR/index.htm, Neurovasculature > Atlas of Brain Arteries (Frontal Section) > Atlas > Slide 15); Fe=0 mg/kg: plain scan without USPIO enhancement; Fe=3 mg/kg: USPIO enhancement scan with a concentration of 3 mg/kg of Fe. USPIO-SWI imaged the LSAs more clearly compared with conventional SWI sequences.

**Figure 3:**
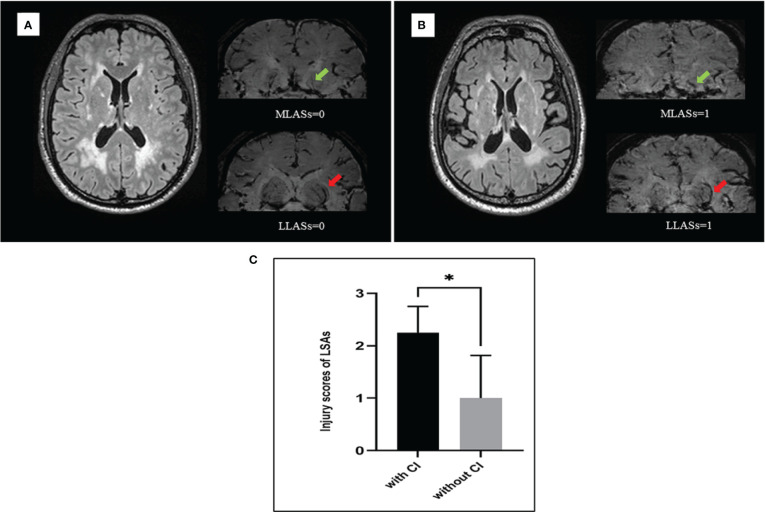
The injury severity of lenticulostriate arteries (LSAs) was different between patients with and without cognitive impairment when under similar white matter hyperintensities (WMH). **(A)** male, 50 years old, had severe WMH and normal cognitive function, the injury scores of medial and lateral LSAs were 0 separately. **(B)** male, 52 years old, had severe WMH and cognitive impairment, the injury scores of medial and lateral LSAs were 1 separately. **(C)** Eight patients with severe WMH were divided into two groups matching for sex, age, and risk factors. One group consists of four patients who had cognitive impairment and another group consists of four patients who had normal cognitive function. The injury scores of LSAs were statistically different between two groups (p=0.04). MLASs, medial lenticulostriate arteries; LLSAs, lateral lenticulostriate arteries; CI, cognitive impairment. *p < 0.05.

To explore the underlying mechanisms associated with heterogeneity of WMH severity on cognitive impairment, we applied USPIO-SWI to visualize the small vessels in the basal ganglia region, where the WMH were apparent and arterioles were the main supplying arteries. We performed USPIO-SWI on eight patients with severe WMH; among them, four had cognitive impairment, and the other four did not. Age, sex, presence of BG-ePVS and microbleeds were similar between the two groups. The imaging of medial and lateral LSAs was shown in **Figure 2**. The injury scores of the penetrating arteries were higher in patients with cognitive impairment than in those without (p<0.04, **Figure 3**). The results suggested that the injury degree of penetrating arteries in the basal ganglia might be responsible for the cognitive impairment in patients with similar severity of WMH, causing WMH heterogeneity in CSVD patients with cognitive impairment.

## Discussion

We demonstrated the heterogeneity of WMH in cognitively impaired CSVD patients by: 1) we observed a “mismatch” between the WMH severity and cognitive impairment. 2) We found that in addition to conventional factors, e.g. age, BG-ePVS, microbleeds and total CSVD score, the injury to penetrating arteries might also be the important contributor to this mismatch, which might increase the risk of cognitive impairment when under similar WMH. These findings can help guide prevention and treatment of cognitive impairment in at-risk CSVD patients with WMH.

CSVD has been associated with impairment in almost all major domains of cognitive ability (23). WMH were the most common imaging feature of CSVD, several studies have demonstrated that a high burden of WMH is associated with a noticeable decline in global cognitive function in both normal and high-risk populations (5). The progression of WMH is associated with further decreases in global cognitive scores (24, 25). Besides, above certain severity, WMH increased the risk of all-cause dementia (26). Our regression analysis were consistent with the findings from previous studies. In addition to WMH, we found that other imaging features of CSVD, including lacunes, ePVS in basal ganglia and microbleeds, were also associated with cognitive impairment, which was in line with the results of recent studies (27).

Heterogeneity of WMH has been reported before. Wardlaw demonstrated the progression of WMH was heterogeneous and the volume of WMH might grow and/or regress, reflected in the change of brain volume, visual scores, and mean diffusivity in WMH (7). Keun-Hwa Jung showed the variation on clinical symptoms related to WMH, that the periventricular WMH were associated with hypertension and deep WMH were associated with poor sleep quality (28). However, the heterogeneity of WMH in cognitively impaired patients has not been reported. In present study, we found a “mismatch” between the WMH severity and the decline in cognitive function. To explore the underlying mechanisms, we compared the vascular risk factors and imaging features possibly contributing to the cognitive dysfunction between patients with and without this mismatch. We found that changes on imaging features of CSVD may be important contributors of cognitive impairment, while traditional vascular risk factors, such as hypertension, diabetes mellitus, smoking, and drinking, did not play a prominent role in this mismatch. Our results suggested that the relationship between WMH severity and cognitive impairment might be independent of vascular risk factors as previously reported (26), and these factors might not show any promise for identifying patients with mismatch type.

Advances in structural and functional neuroimaging have facilitated the exploration of the mechanisms of WMH heterogeneity. Maillard investigated white matter integrity within WMH by using the diffusion tensor imaging (DTI) and found that although WMH had similar appearance on FLAIR, the diffusion metrics were significantly different and each could have different progressions over time (7, 29). In addition, the heterogeneity of white matter integrity might exist not only in WMH but also in penumbral or even remote areas. The variation in functional connectivity between different brain regions might be responsible for various clinical symptoms (11). Furthermore, the heterogeneity of blood-brain barrier (BBB) permeability might explain the mechanisms to some extent. BBB leakage tends to occur in ePVS-WMH or WMH surrounding the lacunes rather than only within WMH (30). Therefore, potential difference in BBB leakage between WMH with ePVS or lacunes and those without might be another reason why similar WMH could be associated with different clinical outcomes.

To further explore the mechanisms underlying the heterogeneity of WMH severity on cognitive impairment, we applied USPIO-SWI to visualize the penetrating arteries in the basal ganglia. USPIO-SWI has previously been used to image small vessels *in vivo* on a subvoxel level with reliable safety and maneuverability (12, 13, 31). Thereinto, E. Mark Haacke were achieved to visualizing the small arteries and veins in midbrain using USPIO-SWI on 3-Tesla MRI, and realized the measurement of the diameter of those small vessels (12). However, few studies have successfully imaged penetrating arteries in the basal ganglia due to the deep and complex anatomical location. We preliminarily visualized the medial and lateral LSAs on 3-Tesla USPIO-SWI and created a visual scale to assess the injury to these vessels. We found differences in numbers of these supplying arteries within WMH, and these differences might be related to the cognitive impairment. In addition to the amount, the length, diameter and stiffness of these supplying arteries might also be important metrics determining the actual severity of WMH, which need further exploration. The injury of penetrating arteries caused by arteriolosclerosis, inflammation, abnormal immunity and other etiological conditions could lead to the cerebrovascular dysfunction, such as the decrease of cerebral vasoreactivity and pulsatility, the disorder of vasomotion and even the luminal stenosis (32). The dysfunction of cerebral vessels could disturb the maintenance of the normal cerebral blood flow (CBF) regulation and BBB, which would result in the accumulation of amyloid-β proteins and impaired drainage of extravascular proteins along blood vessels (33). Furthermore, the dysfunction of CBF might lead to ischemia, which could cause the loss of myelin and axons, as well as the development of gliosis (10), disrupting the connections between cortex and the subcortex nuclei (34). These factors together contribute to the heterogeneity of WMH in clinical symptoms. Although some manual steps during the reconstruction of the penetrating arteries might influence the accuracy of the imaging, and the blooming artifact could affect the true size of these small vessels (12, 13, 31), our study provided great confidence in the utility of exploring the heterogeneity of WMH and the mechanisms of CSVD in the future.

Our study has several limitations. Firstly, the current study was a cross-sectional study and the sample size was relatively small. Larger longitudinal studies are needed to explore the heterogeneity of WMH and the progressive changes in cognitive function to better identify patients at risk of developing cognitive impairment over time. Secondly, although previous studies have shown that a MoCA score of <26 was consistent with cognitive impairment, our study could not determine the specificity and sensibility of the MoCA scores due to lack of formal neuropsychological testing. Depression, apathy or fatigue associated with CSVD might contribute to lower MoCA scores and therefore might reduce the specificity (21). Lastly, the domains of cognitive impairment might be associated with the location of WMH, it could be another factor related to the heterogeneity between WMH and cognitive impairment. Further exploration of the various locations of WMH across different cognitive domains is needed to obtain more robust evidence that the heterogeneity of WMH in cognitive impairment is clinically or physiologically relevant and shows specificity. Despite limitations, this study was innovative since the technique of imaging penetrating arteries in the basal ganglia was utilized in order to explore the mechanisms causing the heterogeneity of WMH.

## Conclusions

Heterogeneity of WMH in cognitively impaired CSVD patients is present. Even though the WMH appeared similar on conventional imaging, the higher degree of injury to the supplying penetrating arteries in WMH or the presence of other CSVD imaging features might be indicative of cognitive impairment. The results of our study provided data to support early identification and prevention of cognitive impairment in patients with WMH. Larger population-based studies are required to determine the various types of heterogeneity of WMH and progression into different domains of cognitive function.

## Data Availability Statement

The original contributions presented in the study are included in the article/supplementary material. Further inquiries can be directed to the corresponding author.

## Ethics Statement

The studies involving human participants were reviewed and approved by the Institutional Review Boards of Beijing Tiantan Hospital. The patients/participants provided their written informed consent to participate in this study.

## Author Contributions

TW was responsible for the study concept and design, clinical data collection, MRI data processing, and manuscript drafting. AJ analyzed the data and generated the figures. YF, ZZ, and SL conducted study design, MRI data acquisition, and interpretation of data. DW revised the manuscript. YW was responsible for the study concept and design, study supervision, analysis and interpretation of data, and clinical revision of manuscript for intellectual content. All authors contributed to the article and approved the submitted version.

## Funding

Our study was funded by grants from the National Natural Science Foundation of China (No. 81825007), Beijing Outstanding Young Scientist Program (No. BJJWZYJH01201910025030), Youth Beijing Scholar Program (No.010), Beijing Talent Project - Class A: Innovation and Development (No. 2018A12), “National Ten-Thousand Talent Plan”- Leadership of Scientific and Technological Innovation, National Key R&D Program of China (No. 2017YFC1307900, 2017YFC1307905) and the National Key Research and Development Program (2018YFC1312400, 2018YFC1312402) from the Ministry of Science and Technology of China.

## Conflict of Interest

The authors declare that the research was conducted in the absence of any commercial or financial relationships that could be construed as a potential conflict of interest.

## Publisher’s Note

All claims expressed in this article are solely those of the authors and do not necessarily represent those of their affiliated organizations, or those of the publisher, the editors and the reviewers. Any product that may be evaluated in this article, or claim that may be made by its manufacturer, is not guaranteed or endorsed by the publisher.
